# Stress in the social environment: behavioural and social consequences of stress transmission in bird flocks

**DOI:** 10.1098/rspb.2024.1961

**Published:** 2024-11-13

**Authors:** Hanja B. Brandl, Damien R. Farine

**Affiliations:** ^1^ Centre for the Advanced Study of Collective Behaviour, University of Konstanz, Konstanz 78457, Germany; ^2^ Department of Collective Behaviour, Max Planck Institute of Animal Behavior, Konstanz 78457, Germany; ^3^ Department of Evolutionary Biology and Environmental Studies, University of Zurich, Zurich 8057, Switzerland; ^4^ Division of Ecology and Evolution, Research School of Biology, Australian National University, Canberra 2601, Australia

**Keywords:** stress transmission, physiological contagion, stress response, social environment, social stress, zebra finches

## Abstract

The stress response helps individuals cope with challenges, yet how individual stress levels shape group-level processes and the behaviour of other group members has rarely been explored. In social groups, stress responses can be buffered by others or transmitted to members that have not even experienced the stressor first-hand. Stress transmission, in particular, can have profound consequences for the dynamics of social groups and the fitness of individuals therein. We experimentally induced chronic stress within replicated colonies of zebra finches and used fine-scale tracking to observe the consequences of stress-exposed colony members for the behaviour and reproduction of non-manipulated colony members. Non-manipulated individuals in colonies containing stress-exposed individuals exhibited reduced activity, and fewer—but more differentiated—social bonds. These effects were stronger in colonies with a greater proportion of stress-treated individuals, demonstrating that the impact of stressors can reach beyond directly exposed individuals by also affecting their group mates. We found no evidence that socially transmitted stress affected reproduction or long-term physiological measurement in unmanipulated birds, even though the stress-exposed demonstrators laid slightly fewer eggs and showed stressor-dependent changes in feather corticosterone. Social transmission of these effects, if occurring at all, might be more subtle.

## 1. Background

Animals are facing increasing numbers of stressors as progressing urbanization and climate change lead to unprecedented rates of environmental change. As most animals spend at least part of their lives in social groups or aggregations, they rarely face these challenges alone. It is well-established that the social environment, comprising social relationships and interactions, affects how individuals interface with their environment [1] as well as their wellbeing and physiology [2]. However, how stress in the social environment interplays with individuals' physiology, their behaviour and the functionality of groups has rarely been explicitly addressed, especially in animals forming differentiated and long-term social relationships. There are two predictions for how stressed individuals (i.e. showing a stress response through activation of the autonomic and neuroendocrine systems; reviewed by e.g. [3–5]) may affect, or be affected, by group mates. First, individuals can buffer the stress responses of others, in which cases the stressed individual benefits from interacting with non-stressed individuals. Second, stress can transmitted between individuals, where interacting with a stressed individual induces a stress response in individuals that have never previously encountered the stressor—i.e. the primary trigger of the stress response—first-hand (reviewed in [6]). While empirical evidence for social buffering is relatively widespread [7–9], empirical evidence for stress transmission is scarcer.

Stress transmission occurs, and is widely studied, in humans [10,11]. In non-human animals, it has been detected in rodent species [12–14] and—at least in the nestling stage—in one bird species [15]. For example, the process of stress transmission is detectable in the brains of mice, where the synaptic responses to experiencing a stressor directly, or to encountering other individuals that have experienced the stressor, are near-identical [13]. In both rodents and nestling birds, exposure to stressed individuals has also been shown to trigger autonomic, endocrine and behavioural responses that are similar to those seen in individuals that have experienced direct stress exposure [12,14,15], suggestive of stress transmission. Yet, the individual-, group- and population-level consequences of stress transmission remain largely unknown.

Stress transmission could have positive or negative impacts in social animals. Matching of physiological (and emotional) states might make it easier to predict the actions of others [16], and can promote social attraction [17] and cooperation [18] in humans. The transmission of stress responses could also serve as information transmission to quickly warn about potential threats in the surroundings [6]. This could help individuals to anticipate the need to avoid or escape from impending dangers, or aid in the selection of new habitats or social groups by generating the ability to avoid high-stress environments. By contrast, stress transmission could have negative consequences, similar to increasing the frequency and duration of stress exposure. In the short term, stress transmission can lead to behavioural changes (as demonstrated by studies showing behavioural changes owing to direct stress exposure (e.g. [19,20])). Ultimately, these changes can have fitness consequences for the individual and scale up to impact entire groups (e.g. by changing social structure [21] or affecting collective behaviours [6]) or populations (e.g. through Allee effects [22]). Particularly in environments characterized by growing numbers of potential anthropogenic stressors [23] and where stress responses are often maladaptive [24], the balance between adaptive and negative consequences of stress transmission could quickly shift to being more costly.

To understand the consequences of stress transmission, it is essential to identify how stress-exposed individuals can trigger outcomes for other group members. There is widespread evidence that behaviour is affected by physiological state [25,26], that the behaviour of individuals shapes collective processes [27] and that these processes feed back to alter individual states [1]. For example, the composition of the group [28], their collective physiological profile [29] and their physiological synchrony [30] can affect collective outcomes [31], such as group cohesion [30] or group movements [32]. Collective physiology [29] can—in turn—shape the performance of group members in fitness-relevant contexts like foraging and reproduction [28,33]. If outcomes (e.g. how cohesive a group is or their home range) also modulate sensitivity or exposure to stressors, then there could be a significant feedback loop between individuals and their social environment [1].

In this study, we quantified the consequences of long-term stress in replicated colonies of zebra finches. Our aim was to quantify the impact that stressors can have not only on directly exposed individuals, but also on their colony members that never experienced the stressor first-hand. Like many other bird species, zebra finches exhibit a wide range of social behaviours like flocking, collective foraging and colonial breeding, presenting interesting opportunities to explore stress transmission and the consequences of stress in social groups. For example, zebra finches synchronize their reproduction with other breeding pairs in their colony [34] and maintain well-differentiated social relationships over time [35,36]. We thus used replicated colonies of zebra finches in which we experimentally induced long-term stress to different proportions of breeding pairs. We then observed the non-manipulated breeding pairs (i.e. the focal birds) of each colony. Specifically, we measured changes in activity (movement and foraging), sociality (weighted degree, coefficient of variation (*herein CV*) in the proportion of time spent sitting in body contact and pair bond strength), reproductive performance (clutch size and latency) and feather corticosterone (*herein CORT*) as physiological parameters. We then compared individuals breeding in colonies with no, few or many stress-treated colony members. We hypothesized that, if stress transmission was occurring among colony members, unmanipulated individuals would show changes in behaviour, reproduction and physiology that align with stress-treated individuals, and that the effect size would increase as the proportion of stress-treated individuals increases. By showing how individuals that are naïve to the actual source of stress show behavioural responses—i.e. that stress transmission has taken place—we highlight a process with the potential to amplify the effect of stressors in social groups, thereby making them more vulnerable to effects of environmental stressors.

## 2. Methods

### (a) Experimental design

We used 96 two-year-old zebra finches that were kept in four separated mixed-sex flocks (12 females and males each) for six months prior to the experiment. Birds had ample time to form pairs and most pairs had bred before (see [36,37]). Flocks were kept in 2 × 2 × 2 m indoor aviaries in separate rooms at 20 ± 1°C with 13L : 11D with a full-spectrum lighting system. Birds were provided with ad libitum access to finch seed mix, and with cucumbers and mineral supplements twice per week. Each flock contained one to two additional pairs for replacing birds between experimental rounds to maintain equal colony sizes.

**Figure 1.**
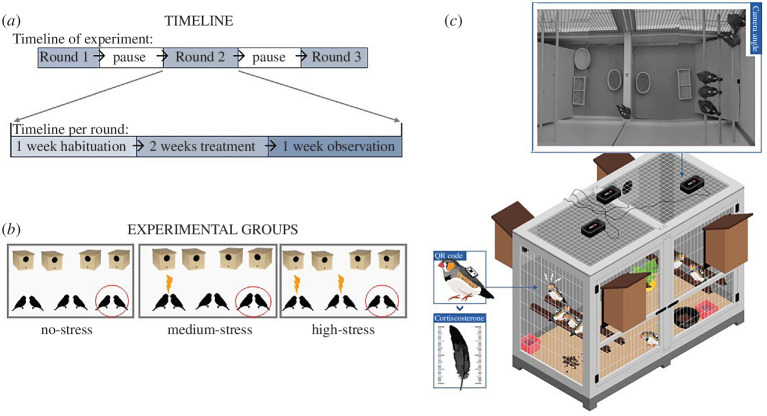
Experimental setup. (*a*) Each experimental round consisted of three periods: one week of habituation, the stress treatment over 14 days, and one additional week of observation of the breeding activity. (*b*) During each experimental round (replicated three times), birds were split up into colonies of three pairs each. Either no pair (no-stress), one pair (medium-stress), or two pairs (high-stress) were exposed to a stress treatment (lightning bolts), with other pairs acting as controls (no highlight) or focal pairs (red circle). (*c*) Cages were designed to facilitate continuous automated tracking of individual movements and social behaviours, made possible by fitting each individual with a unique QR barcode backpack [38]. (Drawing provided by ©Centre for the Advanced Study of Collective Behaviour.)

The experiment was performed in three repeated rounds (figure 1*a*), where each flock was split up into smaller cages (120 × 50 × 100 cm) containing three pairs (i.e. colonies, figure 1*b*). Bonded pairs were kept together throughout the experimental rounds, but the colonies were assembled from three different randomly chosen pairs of the same flock in every round (while keeping the four flocks separate at all times). Birds were kept in the colonies for four weeks per experimental round (figure 1*a*). Each experimental round was followed by a one month pause during which the birds were returned to their original flock. Experimental cages had a social perch on both far ends of the cage (three horizontal bars about 3 cm apart) and a middle perch (one bridge), two full seed-bowls and a water bath, sand and grit (figure 1*c*). Four nest boxes were installed on the outside of each cage four days after the start of the treatment period and left there for 18 days with nest material available.

### (b) Direct stress manipulations (for direct stress effects)

We experimentally induced long-term chronic stress over 14 days. We expected that repeated/lasting stress exposure would have more measurable downstream consequences than individual acute stress responses. We used two different approaches on half of the stress-treated birds each: a behavioural stress protocol and subcutaneous CORT implants. The behavioural stress protocol consisted of stressors applied for 30 mins per day at random times (adapted from [39] to induce chronic psychological stress through the constant anticipation of repeating, yet unpredictable, stressors). Specifically, birds were removed from the cages once per day and, in an adjacent room, were exposed to one of five different stressors (restraint in cloth bag, cage disturbance, cage rolling, loud music or tube restraint; see electronic supplementary material, text for details), rotating daily. Unpredictable challenges can increase CORT levels, reflecting activity of the hypothalamic–pituitary–adrenal (HPA) axis, thereby mediating adaptive physiological and behavioural changes in the organisms (e.g. [40]). The second approach, exogenous CORT administration, is often used in experiments to simulate sustained elevations of the hormone [41,42] without having to simulate an actual stressor—but possibly also altering the HPA axis function and stress reactivity by causing negative feedbacks [41,43]. We implanted time-release pellets (<3 mm diameter; Innovative Research of America) subcutaneously between the scapular through a small incision, releasing a total of 0.25 mg CORT (17.86 µg day^−1^; see electronic supplementary material, supplementary text). Control birds received a sham version of their respective stress treatment type to control for the effects of handling (behavioural stress) and surgical (CORT administration) procedures. We used two experimental approaches to benefit from their relative strengths—the behavioural stress protocol simulates natural stress exposure (despite involving stimuli that would not be encountered in the wild) while the CORT administration avoids the need for further manipulation or interference with colonies for the duration of the treatment. At the same time, any differences between the results of the treatments could give additional insights into the mechanisms (i.e. whether an effect is driven mostly by CORT).

### (c) Social stress treatments (for socially transmitted stress effects)

Each colony was assigned to either no-stress (without stress-treated birds), medium-stress (with one stress-treated pair) or high-stress (with two stress-treated pairs; figure 1*b*). In each experimental round, each pair was pseudo-randomly (to avoid repetition) assigned to one colony, and therein, to one treatment: focal, control or stress. Focal birds remained completely untreated, only varying in the number of stress-treated neighbours in their colony, which allowed us to measure effects of socially transmitted stress on them. Colonies of different treatment types (behavioural/pharmacological) were kept in separate rooms. Colonies in the same room could communicate acoustically but had no visual contact. Partners in a pair always received the same treatment. Across experimental rounds, each bird was stress-treated once (either behaviourally or pharmacologically), received a sham treatment once (control) and acted as a focal once. Thus, over the three repeated rounds of the experiment, all 96 birds received a stress treatment once—48 of them as behavioural stress treatment and 48 of them as CORT implant (see electronic supplementary material).

**Figure 2.**
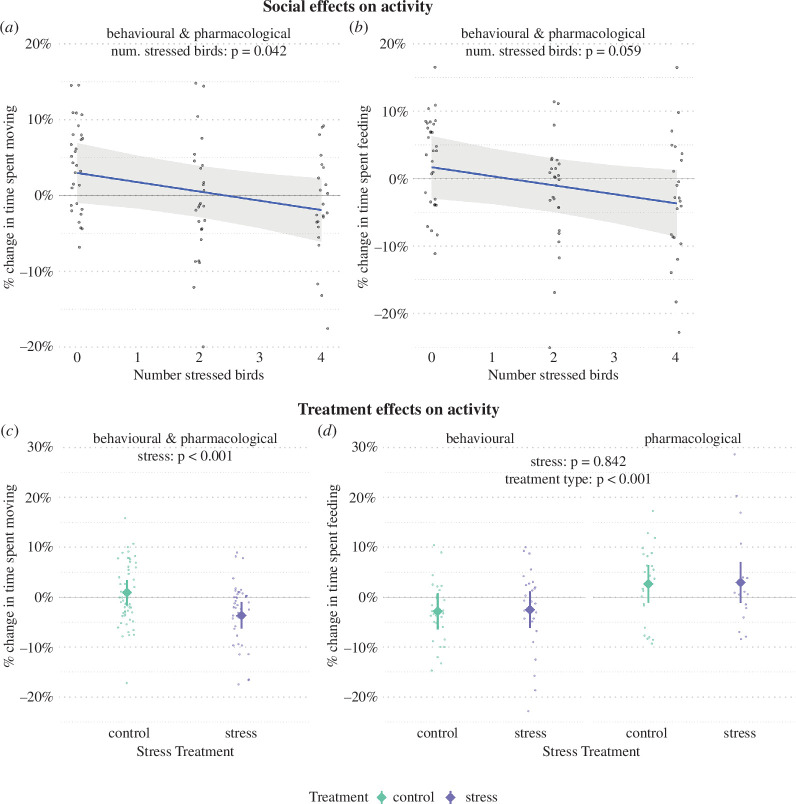
Predicted changes in the percentage of time spent moving (*a,c*) and time spent feeding (*b,d*) in focal (non-treated) birds in colonies with different numbers of stress-treated birds (top) and in response to the direct stress treatment (bottom). Focal birds had a greater decrease in the percentage of time spent moving between areas of the cage (*a*) and the percentage of time feeding spent feeding (*b*) when more colony members were stress-treated. The decreases represent reduced activity when exposed to stress-treated colony members relative to the period prior to the treatment. Stress-treated birds also showed greater reductions in their time spent moving than the control individuals did (*c*), but there were no discernible differences in how much stress-treated and control birds changed in terms of feeding (*d*). However, changes in feeding differed between treatment types, with the behavioural treatment eliciting a decrease in feeding and the pharmacological eliciting an increase in feeding. Treatment type was not identified as a significant predictor in other models. Predictions are taken from LMMs, with shaded ribbons and error bars showing 95% confidence intervals. Small dots show the raw data, which are jittered to reduce overlap.

### (d) Automated behavioural tracking

Birds were individually marked with a 1 × 1 cm machine-readable barcode mounted on backpacks (~0.2 g) fixed with an elastic harness around their bodies [38; figure 1*c*]. Three RaspberryPi computers with cameras on top of each cage (figure 1*c*) tracked birds in each cage once per second. The pinpoint library [44] extracted the position and orientation of every barcode in the image, from which we could infer movement and social interactions. We then quantified activity (proportion of time spent moving and proportion of time feeding) and sociality (weighted degree, CV and pair bond strength) from these data (calculations described in the electronic supplementary material, supplementary text).

To monitor breeding, we logged nestbox visits using radio frequency identification (RFID) antennas fitted to the entrance of each nest box and attached to RFID decoders. Birds were fitted with a passive integrated transponder tag glued into their backpacks. From these data, we could infer breeding initiation and nest attendance. We also conducted in-person observations once per day, to collect data on how many eggs were laid by the female in each pair.

### (e) Feather corticosterone

To assess endocrine effects of the stress treatments, we quantified the amount of CORT deposited in the tail feathers grown before experimental rounds and during the two weeks of treatment. Measuring feather CORT can estimate HPA-axis activity over longer time periods because CORT is deposited from the blood stream into growing feather tissue over the whole period of feather growth [45–47] and this method is also less invasive than evaluating blood hormone levels. The feathers grown before (mean length: 40.4 mm ± 5.0 s.d.) and during (mean length: 20.0 mm ± 4.8 s.d.) the experiment were processed using the ‘Hair, Feather and Nail Extraction Protocol’ provided with the ELISA kit (enzyme-linked immunosorbent assay; DetectX^®^; K014-H1W; Arbor Assay) and the methodology of Bortolotti *et al*. [47], with some modifications (see electronic supplementary material, supplementary text for details).

After extraction, feather CORT concentrations were quantified following the manufacturer’s protocol, by measuring absorbance at 450 nm (which is inversely proportional to CORT concentration) using a microplate absorbance reader (iMark, BioRad; see electronic supplementary material, supplementary text for details). Intra-plate CV was 2.92% and inter-plate CV obtained from a high- and low-concentration pooled sample was 16.36% (*n* = 8 plates; 5 additional plates were run without pools). We obtained measurements of 404 samples from 95 individuals, and used 305 of them in the following analysis after removing values (*n* = 81 samples) where the percentage bound (B/B0) was outside the range of parallelism with the standard curve (>90% or <20%), and 18 outliers with values >1.5 × interquartile range. Removing outliers substantially improved model fit and we cannot say with certainty whether those values were biologically meaningful or inflated owing to small sample amounts [48]. The sample size used in the models was further reduced because we only included complete sets where before and after values were both available.

### (f) Statistical analysis

First, we analysed whether the number of stress-treated colony members affected activity (percentage of time moving, and feeding), social behaviour (weighted degree, CV, pair bond strength), reproduction (clutch size, clutch latency) and physiology (feather CORT) in unmanipulated focal birds. For each of these eight response variables, we fit a linear mixed effect model (LMM) with the number of stress-treated birds in their environment (0–4), treatment type (behavioural versus pharmacological), treatment round (round 1, 2 or 3) and sex (for individual-level models) as predictors (see model structure in electronic supplementary material, figure S1*a*). We used AIC model selection to decide whether to include treatment type as a main effect and as an interaction with the number of stress-treated birds in the model because we did not know *a priori* if we should expect differences between the treatment types. Random effects included pair ID (an identifier of each pair to prevent pseudoreplication across rounds) and flock ID (four flocks of zebra finches were used) in all models. Random effects were dropped whenever the variance components were estimated as zero [49].

Second, we tested the direct effect of the stress treatment on birds. Here, we used the same model structure as above, but compared control birds from control cages with stress-treated birds from the high-stress environment—the colonies where we expected the see the most pronounced difference and less effects of stress transmission (electronic supplementary material, figure S1*b*). We again used AIC model selection to decide whether to include treatment type (behavioural or pharmacological) as an additional predictor in the models (as a main effect or as two-way interaction with treatment). For the analyses using pair-wise data (pair bond strength and breeding), we also included the low-stress environments to contrast against the no-stress cages for assessing the direct stress effects because of the reduced sample size.

The response variables for both sets of models were obtained as follows. For activity and social variables, we calculated the change in behaviour between the time before and during the treatment. We defined three days before the onset of the treatment (days 4–6 in colonies) as the pre-phase and the first three days after the onset of the treatment (days 8–10) as the treatment phase. We did not include the period during which the nest boxes were installed on the cages (days 11–28) in the tracking-based analyses because birds engaging in nest building and incubating were less visible to the cameras. Nevertheless, we evaluated the reproductive effort during this time. For feather CORT, we used the difference in the measurements from the feathers collected before and after each round, in pg CORT per mm feather.

For activity, the social variables and physiological measurement, we fit models with the Gaussian error family (using lme4 [50]). For clutch size and clutch latency, we fit generalized mixed models (GLMMs) with a compois (Conway–Maxwell Poisson distribution) and a Poisson regression with glmmTMB [51] to better handle under-dispersed Poisson data. Model fits were assessed using QQ-plots and diagnostic plots generated with Dharma [52]. Finally, we fit one additional binomial model to demonstrate that the propensity to lay eggs generally did not differ between treatments, including the same covariates and random effects as above.

All statistical analyses were performed using the R software [53]. In the figures, treatment types are shown separately if there was at least weak statistical support for a difference between them. All data and code to replicate the analyses are available [54].

## 3. Results

### (a) Effects of socially transmitted stress on activity levels

Focal birds (i.e. birds that did not receive any direct manipulations) decreased their movement during the treatment period by on average 1.23% for every stress-treated bird in its social environment (figure 2*a*, table 1). For a focal bird in a high-stress colony containing four stress-treated members, this corresponded to an estimated decrease of 4.90% in time spent moving compared with a bird in a no-stress cage. We found an effect in the same direction when comparing stress-treated birds of the high-stress cages with the control birds from the no-stress cages to evaluate the direct effect of the stress treatment (stress treatment: *β* = −4.59, *t* = −4.19, *p* < 0.001, *n* = 97; electronic supplementary material, figure S1*d*, table S1). We found no support for movement changing differently between the sexes and no difference in socially transmitted stress effects between experimental rounds (table 1), but there was an additional decrease in movement from round 1 to round 2 for the direct stress effect (round 2: *β* = −4.62, *t* = −3.13, *p* = 0.002; electronic supplementary material, table S1).

**Figure 3.**
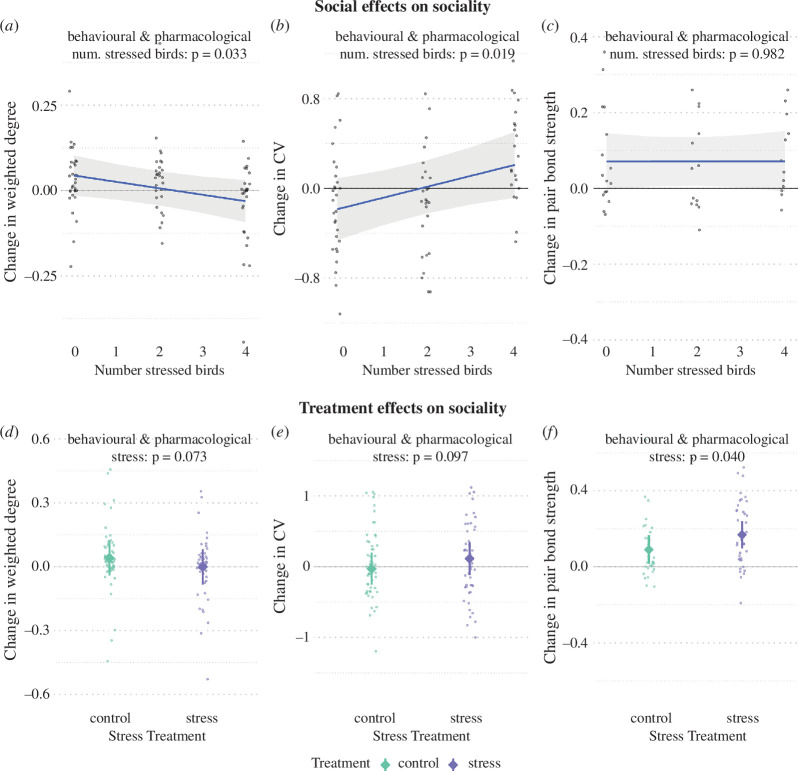
Predicted changes in social behaviour during the treatment period in focal (non-treated) birds in colonies with different numbers of stress-treated birds (*a*–*c*) and in response to the direct stress treatment (*d*–*f*). Weighted degree decreased (*a*) and the CV increased (*b*) between the pre-treatment and during-treatment periods in focal birds in colonies with more stress-treated birds. The direct effect of the stress treatment (comparing stress-treated birds with control birds) showed a similar direction of effects for weighted degree (*d*) and CV (*e*). Predictions are taken from LMMs, with the shaded ribbon showing 95% confidence intervals. Small dots show the raw data, jittered to reduce overlap.

**Table 1.** Effect of stress in the social environment on the change in the percentage of time spent moving (left) and feeding (right) of untreated focal birds in colonies containing different numbers of stress-treated birds. Gaussian linear mixed-effect regression model evaluating the effect of the number of stress-treated birds in the social environment on the change in the percentage of time moving and feeding. Bold values denote statistical significance at the *p* < 0.05 level.

	social effects on change in percent of time moving					social effects on change in percent of time feeding				
predictors	estimate	s.e.	*t*	CI	*p*	estimate	*s.e.*	*t*	CI	*p*
(intercept)	1.18	2.09	0.56	−3.04 to 5.40	0.577	1.02	2.47	0.41	−3.96 to 6.00	0.681
numStressBirds	−1.23	0.58	−2.11	−2.41 to −0.04	**0.042**	−1.34	0.68	−1.96	−2.73 to 0.05	0.059
round (2)	3.80	2.38	1.59	−1.04 to 8.63	0.120	0.42	2.81	0.15	−5.28 to 6.12	0.882
round (3)	−0.69	2.32	−0.30	−5.41 to 4.02	0.768	−2.18	2.73	−0.80	−7.74 to 3.37	0.430
sex (m)	1.54	1.06	1.46	−0.60 to 3.68	0.153	2.51	1.27	1.98	−0.07 to 5.09	0.056
**random effects**										
*σ* ^2^	21.19					30.75				
*τ*_00_	25.17 pairID					34.11 pairID				
*N*	42 pairID					42 pairID				
observations	79					79				
marginal *R*^2^/conditional *R*^2^	0.172/0.622					0.120/0.583				

We also found some evidence that focal birds decreased their feeding activity by 1.34% per stress-treated bird in their environment (figure 2*b*, table 1), although we found no evidence of a decrease in feeding activity when comparing stress-treated individuals with control birds (stress: *β* = 0.30, *t* = 0.20, *p* = 0.842, *n* = 97; figure 2*d*, electronic supplementary material, table S1). However, the direct treatment effect model showed that implanted birds (both CORT and placebo birds) expressed a 5.48% greater increase in feeding relative to the behavioural treatment group (pharmacological treatment: *β* = 5.48, *t* = 3.54, *p* = 0.001; electronic supplementary material, figure S2*d*, table S1). We also found evidence that males overall spent more time feeding than females, but detected no difference in feeding activity between experimental rounds (table 1 and electronic supplementary material, S1).

### (b) Effects of socially transmitted stress on social behaviours

The number and strength of a focal bird's relationships (weighted degree) with colony members (excluding their partner) decreased more in colonies with more stress-treated colony members. Changes (from before to during the treatment) ranged from a slight increase in no-stress cages (+0.04 weighted degree) to no change in low-stress cages to a marked decrease in high-stress cages (−0.03; figure 3*a*, table 2). The stress-treatment effect on changes in weighted degree was also negative (when compared with changes in control birds; stress: *β* = −0.04, *t* = −1.81, *p* = 0.071, *n* = 114; electronic supplementary material, figure S3*d*, table S2).

**Table 2.** Effect of stress in the social environment on the change in weighted degree, CV and pair bond strength of untreated focal birds in colonies containing different numbers of stress-treated birds. Gaussian linear mixed-effect regression model evaluating the effect of the number of stress-treated birds in the focal birds' social environment on the change in the number and strength of relationships with individuals other than their mated partner (weighted degree), on the change in the CV (with higher values indicating more differentiated relationship) and on the change in the strength of the pair bond with their mated partners. Bold values denote statistical significance at the *p* < 0.05 level.

social effects on change in weighted degree						social effects on change in CV				
predictors	estimate	s.e.	*t*	CI	*p*	estimate	s.e.	*t*	CI	*p*
(intercept)	0.04	0.03	1.40	−0.02 to 0.11	0.168	−0.26	0.14	−1.86	−0.55 to 0.02	0.071
numStressBirds	−0.02	0.01	−2.21	−0.04 to −0.00	**0.035**	0.10	0.04	2.47	0.02 to 0.18	**0.019**
round (2)	−0.02	0.04	−0.58	−0.09 to 0.05	0.566	0.16	0.16	0.98	−0.17 to 0.48	0.332
round (3)	0.05	0.03	1.43	−0.02 to 0.12	0.163	0.13	0.16	0.77	−0.20 to 0.46	0.444
sex (m)	−0.02	0.02	−1.06	−0.05 to 0.02	0.296	−0.04	0.09	−0.42	−0.21 to 0.14	0.676
**random effects**										
*σ* ^2^	0.01		0.14							
*τ*_00_	0.01 pairID		0.09 pairID							
*N*	39 pairID		39 pairID							
observations	86		76							
Marginal *R*^2^/conditional *R*^2^	0.137/0.548		0.118/0.464							

**s**ocial effects on change in pair bond strength										
predictors	estimate	s.e.	*t*	CI	*p*					
(intercept)	0.02	0.04	0.56	−0.06 to 0.10	0.579					
numStressBirds	0	0.01	0.02	−0.02 to 0.02	0.986					
round (2)	0.08	0.05	1.69	−0.02 to 0.17	0.099					
round (3)	0.07	0.05	1.46	−0.03 to 0.16	0.151					
observations	41									
marginal *R*^2^/conditional *R*^2^	0.082/0.007									

As relationships weakened, they also became more differentiated for the focal birds living in higher-stress environments, as reflected by an increase in the CV (figure 3*b*, table 2). This change for focal birds within stress-treated colonies matched the direction of the stress treatment (i.e. stress-treated birds increasing CV more than control birds; stress: *β* = 0.07, t = 1.68, *p* = 0.097, *n* = 106; see figure 3*e* and, electronic supplementary material, table S2). We found no support for a difference between the sexes or experimental rounds in how social relationships changed in response to socially transmitted or direct stress (table 2 and electronic supplementary material, S2).

Changes in the strength of pair bonds (edge weight) between the males and females of mated focal bird pairs did not differ with the proportion of stress-treated members in their environment (figure 3*c*, table 2). However, we did find support for a greater increase in pair bond strength in pairs of stress-treated birds relative to control birds (stress: *β* = 0.08, *t* = 2.11, *p* = 0.040, *n* = 71; figure 3*f*, electronic supplementary material, table S2). Pair bonds generally became stronger with every experimental round (round 2: *β* = 0.16, *t* = 3.47, *p* = 0.001; round 3: *β* = 0.17, *t* = 3.63, *p* = 0.001; electronic supplementary material, table S2).

### (c) Effects of socially transmitted stress on reproduction

We found no evidence that reproduction was affected by stress in the social environment in our experiment. The number of stress-treated colony members did not affect the clutch sizes of the untreated focal pairs (electronic supplementary material, figure S2*a*, table S3). Stress-treated pairs in high-stress colonies laid on average −0.67 less eggs than no-stress control pairs (electronic supplementary material, figure S2*c*, electronic supplementary material, table S3).

We also found no evidence that the number of stress-treated pairs in a colony affected the timing of when pairs started to lay eggs (electronic supplementary material, figure S2*b*, table S3). The model evaluating the direct effect of the stress treatment gave weak support for an increase in latency to lay eggs by 0.75 days following a stress treatment (stress: IRR = 1.14, *z* = 1.69, *p* = 0.091, *n* = 61; electronic supplementary material, figure S2d, table S4) and strong statistical support for birds receiving the behavioural table treatment laying eggs 1.79 days later than those having received implants (pharmacological: IRR = 0.73, *z* = −4.02, *p* < 0.001; electronic supplementary material, figure S2*d*, table S34). The propensity of pairs to initiate egg laying did not differ between the treatments (stress: log-odds = 1.81, *z* = 0.83, *p* = 0.406, *n* = 71; electronic supplementary material, table S5).

### (d) Effects of socially transmitted stress on feather corticosterone

**Figure 4.**
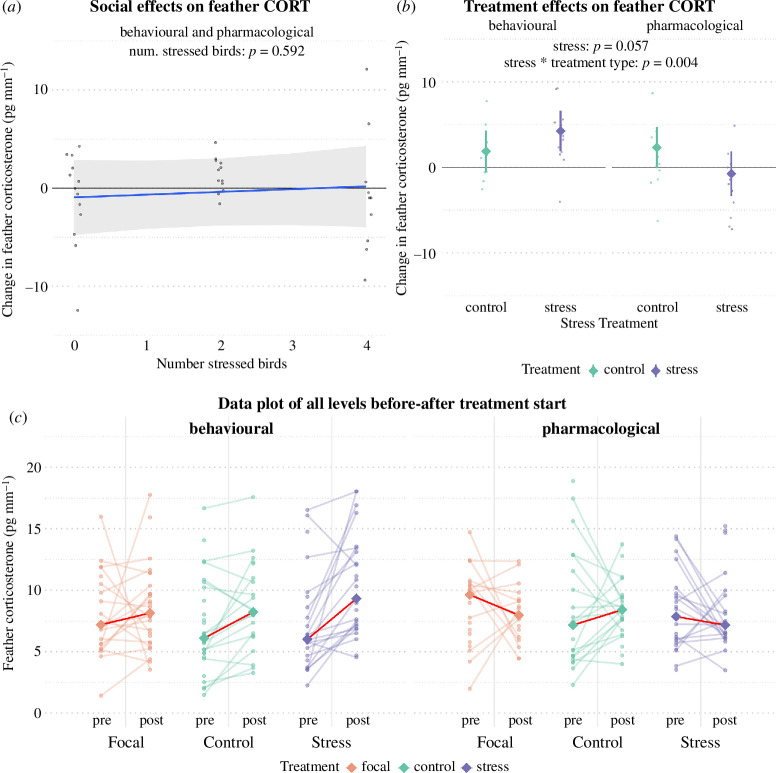
(*a*) Predicted change in feather CORT concentrations in focal (non-treated) birds in colonies with different numbers of stress-treated birds, (*b*) change in feather CORT in response to the direct stress treatment versus control and (*c*) raw data plots of all measured feather CORT concentrations across the three treatment groups. In (*a*), mean feather CORT levels did not change with different numbers of stress-treated birds in their colony. In (*b*), feather CORT levels changed differently in response to behavioural (left) or pharmacological (right) stress treatment (purple) compared with control (green). The shaded ribbon (*a*) and error bars (*b*) show 95% confidence intervals for the predicted means obtained with GLMMS, with dots showing the raw data (*a,b*). (*c*) Raw measurements of feather CORT before and after the treatment period, with coloured lines connecting measurements of the same individuals and with diamonds giving the mean values for the respective treatment group.

We found no support in the data for focal birds' feather CORT levels being affected by the number of stress-treated birds in their colony (number stressed birds: *β* = 0.28, *t* = 0.55, *p* = 0.592, *n = 35;* figure 4*a*, electronic supplementary material table S6). We found some evidence that the stress treatments itself did affect feather CORT levels in the stress-treated birds (stress: *β* = 2.37, *t* = 1.96, *p* = 0.057, *n* = 47), but differently depending on treatment type—birds exposed to the behavioural protocol increased feather CORT compared with pre-treatment values, while the CORT levels of pharmacological treatment birds with implants dropped to below pre-treatment values (stress × pharmacological treatment: *β* = −5.45, *t* = −3.02, *p* = 0.004; figure 4*b*, electronic supplementary material, table S7).

We found that males of the untreated focal birds increased their feather CORT levels (from before to after the treatment period) more strongly than females, by on average 3.73 pgmm^−1^ feather (males: *β* = 3.73, *t* = 2.78, *p* = 0.013; electronic supplementary material, table S6). Stress-treated males, however, decreased feather CORT values from before to during the treatment more than females (males: *β* = −2.18, t = −2.57, *p* = 0.014, *n* = 47; electronic supplementary material, table S7). We also found evidence that feather CORT in stress-treated and control birds changed more negatively in rounds 2 (round 2: *β* = −5.14, *t* = −3.94, *p* = <0.001) and 3 (round 3: *β* = −4.65, *t* = −3.24, *p* = <0.001; electronic supplementary material, table S7) compared with round 1. The raw data plot (figure 4*c*) shows all values (including incomplete before–after sets).

## 4. Discussion

In this study, we tested how unmanipulated zebra finches (i.e. the focal birds) respond to having stressed colony members. We found that activity and social behaviours were substantially affected by the social environment. Changes in time spent moving, the rate of social contacts with other colony members and the social differentiation of relationships were all affected by the colony's proportion of stress-treated members. Importantly, these effects were consistently in the same directions as those expressed by stress-treated birds (the direct stress treatment effects). Thus, our results are in line with the predictions of stress transmission, where individuals that are directly exposed to stressors activate parallel stress responses in other individuals that are not directly exposed to the stressor [6]. Our results demonstrate how stress transmission can affect the dynamics and performance of collectives and highlight the importance of considering the consequences of stressors beyond directly affected individuals.

We found relatively few differences between the colonies with birds exposed to behavioural stress and the pharmacological (CORT implants) treatment. While direct stress effects differed for feeding activity, clutch latency and feather CORT, we found no differences between treatment types in the socially transmitted responses. This suggests that the socially transmitted response might be more generalized to different types of stressors and is probably largely CORT-mediated. We used both stress treatment types because each had key advantages, and combining them yielded greater insights into the mechanisms of stress transmission by evaluating similarities and differences in the results. The pharmacological approach using the implants allowed us to leave the birds without any human interference (other than caretaking by experienced staff naïve to the experiment) for the whole 14 days of the treatment. Thus, for the socially transmitted effects that we find in this treatment group, we can rule out that differences among individuals or colonies were caused by the process of administering the treatment. The effects in this treatment group that are proportional to the number of individuals with CORT implants in their cages can thus be clearly ascribed to the pharmacological manipulation of conspecifics and their consequent changes owing to this treatment. With the behavioural stress protocol, we still had the factor of human presence, even though we were very careful to not introduce biases and remained naïve to the exact experimental group assignments during the manipulations. Overall, the similarity in results across the behavioural measurements shows that a lot of the socially transmitted effects are likely to be mediated by CORT, regardless of whether its origin was exogenous or endogenous.

Untreated focal birds and directly stress-treated birds in high-stress colonies spent less time moving than focal birds and control birds in no- or low-stress colonies. The direction of the effect, i.e. a decrease in activity, was in line with our expectations, as CORT administration in birds had been shown to increase perch hopping in the short term [55]—as an acute stress response—but decrease it during longer term exposure [56]. In the wild, decreases in activity could, for example, reduce exploration of the environment and home range size, which can restrict access to resources. The time spent feeding was also reduced in response to socially transmitted stress, but we did not find a direct stress treatment effect there. It is important to note that the control birds on which we assessed the direct treatment effect did receive a control treatment (sham catching or placebo implants), while the focal birds on which we evaluated the socially transmitted stress effects did not receive any external manipulation. Thus, control birds receiving a control treatment might have potentially also experienced more direct stress than focal birds did. Furthermore, it was not possible to measure the direct stress treatment effect completely isolated from a social environment because keeping a zebra finch in isolation from others would constitute a strong stressor itself [57]. That said, foraging is a highly social activity in zebra finches [35] so it is not necessarily surprising that the social environment plays an important role here, and stress transmission and behavioural synchronization might scale up and exaggerate small effects that would not be significant at the individual level.

In the colony social networks, the weighted degree decreased both as direct and as socially transmitted stress effects in the focal birds—the latter with stronger statistical support. Individuals decreased the number and strength of relationships and also differentiated them more—i.e. having a mix of stronger and weaker relationships (a higher CV)—in response to direct and socially transmitted stress exposure. It is generally possible that changes in social structure are linked to the reduction in movement. However, even when not moving, birds were still free to choose who to sit next to and, in that case, we would have rather expected to see a decrease in CV (i.e. more equal associations with all flock mates). A study in voles showed that stress impairs the formation of new relationships, but does not affect relationships with established social partners [58]. It is plausible that zebra finches under stress also sacrificed their weaker associations to focus on their stronger social partners. However, we only found an increase in pair bond strength as a direct effect in the stress-treated birds and not as a socially transmitted stress effect in focal birds. Nevertheless, our findings suggest that stress in the social environment drives a redistribution of social focus towards stronger, pre-existing social partners and away from other colony members. Overall, this means that stress in a social group could reduce cohesion, and, at the same time, a reduction in weaker bonds could lower the chance of stress transmission along these social connections.

Clutch size was reduced owing to the direct stress treatment, which is in line with the general understanding that stress can suppress or negatively impact reproductive functions (e.g. [59]). Reproduction in zebra finches can be deteriorated by stress, e.g. induced through fasting, by increasing CORT, reducing testosterone and reducing courtship behaviour [60]. However, the effects of stressors on breeding appear to be context-dependent, as another study found reduced breeding performance in response to environmental stress in young zebra finches but increases in reproduction when older birds were exposed to the same stimulus [61]. Thus, the lack of statistical support for an effect of socially transmitted stress on the clutch size of focal birds could either be because the effect was too weak to be detected (which is plausible as the effect is in the same direction as that following direct stress), or because birds might respond differently in different contexts. The fact that stressors can have different consequences (including positive effects) depending on timing of exposure and the state of the individual is particularly interesting in the context of stress transmission, as it suggests that contagion of stress responses could have positive consequences beyond its potential role in information transmission. Furthermore, if not all individuals respond in the same way, this can have consequences for the transmission pathways, such as interrupting transmission and preventing stress from spreading further [6]. Finally, the latency to lay eggs was only weakly affected by the direct stress treatment, with the main difference being that the pharmacologically treated birds bred earlier. CORT can facilitate the onset of active breeding [62], but placebo-implanted birds were on average the earliest to lay eggs. Pharmacologically treated colonies were also visited less often by the experimenters. It is thus possible that the control pairs of the behavioural treatment group were also disturbed by the additional daily visit, even though all birds were used to daily care-taking visits.

Interestingly (but not entirely surprisingly), we did not find a socially transmitted effect on the CORT deposited in the feathers during the treatment, and thus cannot show clear evidence that the presence of stress-induced conspecifics triggered the activation of the HPA-axis. There could be multiple explanations for this; for example, feathers may integrate CORT levels over too coarse a timescale for the expected responses to be detected [63]. During chronic stress exposure, plasma CORT levels usually show a dynamic response over time, with periods of elevated baseline CORT, acute stress responses with elevated CORT levels, and a decrease in baseline CORT and damped stress responses following negative feedback of the HPA axis [39,41]. Only if the stressor then still persists will the negative feedback eventually stop and the negative effects of chronic CORT exposure come into play [4]. We currently have no good understanding of how stress transmission interacts with this dynamic of chronic stress, i.e. if transmission might only occur after a certain physiological threshold is reached (thereby perhaps introducing a time lag in response to the stressor), or if it requires enough colony members to respond and for these to respond strongly enough (i.e. complex contagion). Activation thresholds could help avoid costly overreactions and too frequent activation of the stress response. Our study can only represent one puzzle piece, and more research is required to investigate these aspects in more targeted experiments.

A further reason for the lack of socially transmitted effects on feather CORT could be sample size. Unlike behavioural effects, our analyses on feather CORT were limited to 35 data points for social-transmitted stress across both treatment types (21 behavioural and 14 hormonal). This is because some feathers were too small to obtain a sufficiently large sample, and we only used complete before–after feather pairs in the analyses. CORT can inhibit feather growth [64], and birds with high CORT levels were possibly disproportionately missing from the analysis because their feathers did not grow long enough. We nevertheless chose this approach over, for example, taking plasma samples, as we wanted to avoid interfering with individuals’ stress levels by measuring them during behavioural assessment. Finally, it is possible that behavioural synchronization took place that led to individuals copying the stress behaviour of others, with the colony acting more and more like the stress-treated individuals when these were more numerous, without synchronizing their hormonal profiles. However, this scenario is perhaps less likely, given the studies across different species that have shown activation of the endocrine system following similar paradigms, by using more fine-scale hormonal measurements [12,13,15,65].

Finally, we also found differences between the experimental rounds for some measurements of direct stress response, but none for socially transmitted stress. However, since they do not follow a clear pattern, we concluded that these likely stem from not having the treatments balanced completely equally across all rounds. We had to exclude CORT birds from the first round of treatment because they were affected by a gastrointestinal infection (see electronic supplementary material, supplementary text). This could also explain why there was an additional reduction in time spent moving from rounds 1 to 2. However, the other variables did not change in the same way, making it difficult to find a uniform explanation. We included treatment round as a covariate in all models to take these differences into account. Given that individuals went through the treatments in different orders, and birds had six weeks to recover between treatments, we think that it is unlikely that these differences are the result of carry-over effects from previous treatments. Nevertheless, we cannot rule out that stress exposure (direct or socially transmitted) had lasting effects by changing individuals' responses in future stress encounters (e.g. [66]). Future studies could be designed to target the role of carry-over effects in stress transmission. We also did not explicitly test for a role of social buffering of stress, and either or both processes could co-occur. We would have expected social buffering to be more likely to occur in low-stress environments, where we might have seen dampened stress responses—which was the reason why we only contrasted high-stress and no-stress environments for the direct stress effect. Nevertheless, the buffering effect in our study would have probably been subtle and the nuances hard to detect because stress-treated individuals were always together with their social partner, which was also stress-treated. We want to acknowledge these limitations in our experiments that we had to accept in trade-off for other aspects, e.g. optimizing the design to find evidence for stress transmission rather than social buffering, using every individual in every role to control for individual differences, and testing the effects using different stress manipulations to gain insight on the mechanisms of transmission. Ultimately, together with the relatively low number of studies exploring stress transmission in animals to-date, our study highlights how many questions remain open on this topic.

## 5. Conclusion

Our study provides experimental evidence for the strong and widespread impacts that stressed group members can have on the behaviours of other individuals in their social group, through a process that is described as stress transmission. The study of the impact of stressors on wildlife, and also in agricultural systems, has increased over the recent years, and it is becoming evident that considering social components is crucial to understanding when the effects of stressors might scale up and where social groups might be able to provide resilience.

## Data Availability

The data and code used for the analyses are available here [54]. Supplementary material is available online [67].
